# Proton pump inhibitors are associated with increased risk of development of chronic kidney disease

**DOI:** 10.1186/s12882-016-0325-4

**Published:** 2016-08-03

**Authors:** Pradeep Arora, Anu Gupta, Mojgan Golzy, Nilang Patel, Randolph L. Carter, Kabir Jalal, James W. Lohr

**Affiliations:** 1 Department of Medicine, University at Buffalo, 3495 Bailey Ave, Buffalo, NY 14215 USA; 2 Division of Nephrology at VA Medical Center, Buffalo, NY USA; 3 Department of Biostatistics, University at Buffalo, Buffalo, NY USA; 4 Department of Medicine, Virginia Commonwealth University, Richmond, VA USA

**Keywords:** Chronic kidney disease, Death, Outcomes, Proton pump inhibitors

## Abstract

**Background:**

Acute interstitial nephritis secondary to proton pump inhibitors (PPIs) frequently goes undiagnosed due to its subacute clinical presentation, which may later present as chronic kidney disease (CKD). We investigated the association of PPI use with the development of CKD and death.

**Methods:**

Two separate retrospective case–control study designs were employed with a prospective logistic regression analysis of data to evaluate the association of development of CKD and death with PPI use. The population included 99,269 patients who were seen in primary care VISN2 clinics from 4/2001 until 4/2008. For evaluation of the CKD outcome, 22,807 with preexisting CKD at the first observation in Veterans Affairs Health Care Upstate New York (VISN2) network data system were excluded. Data obtained included use of PPI (Yes/No), demographics, laboratory data, pre-PPI comorbidity variables.

**Results:**

A total of 19,311/76,462 patients developed CKD. Of those who developed CKD 24.4 % were on PPI. Patients receiving PPI were less likely to have vascular disease, COPD, cancer and diabetes. Of the total of 99,269 patients analyzed for mortality outcome, 11,758 died. A prospective logistic analysis of case–control data showed higher odds for development of CKD (OR 1.10 95 % CI 1.05–1.16) and mortality (OR 1.76, 95 % CI 1.67–1.84) among patients taking PPIs versus those not on PPIs.

**Conclusions:**

Use of proton pump inhibitors is associated with increased risk of development of CKD and death. With the large number of patients being treated with proton pump inhibitors, healthcare providers need to be better educated about the potential side effects of these medications.

## Background

Proton pump inhibitors (PPIs) became available in the United States in 1989, and have become one of the most widely prescribed classes of drugs. The approved indications for use of PPIs include gastro esophageal reflux disease (GERD), peptic ulcer disease and erosive esophagitis [1]. However, PPIs are often prescribed outside of their approved uses. It has been estimated that up to two-thirds of all people on PPIs do not have a verified indication for the drug. A 2009 analysis using the National Ambulatory Medical Care Survey found an increase in the frequency of prescription PPI treatment from less than five prescriptions per 1000 GERD-related physician visits in 1995 to 43.9 prescriptions per 1000 visits in 2006 in the United States, a more than eight-fold increase [2]. There were a total of 119 million U.S. prescriptions filled in 2009 for all PPIs totaling $13.6 billion [3].

Several studies have shown increased hazards of cardiovascular disease and death with PPIs [4–6]. Renal side effects of PPIs are less often reported and may go unrecognized. These include acute interstitial nephritis (AIN), hyponatremia and hypomagnesemia. In general, most patients with acute kidney injury are assumed to have acute tubular necrosis. It is not surprising that AIN secondary to PPI use may also go undetected due to several reasons: 1) awareness that PPIs can cause AIN may not be wide spread; 2) the time interval from drug initiation to onset of clinical abnormalities is quite variable, ranging from 1 week to 9 months (median 9.9 weeks) [7]; and 3) typical features of hypersensitivity reaction are present in only a minority of cases. Patients with undiagnosed AKI due to PPIs may present later with chronic kidney disease (CKD). However, data is lacking on the association of PPI use with CKD. In this manuscript we investigate the relationship of PPI use and the development of CKD and death.

## Methods

### Study design and setting

Two separate retrospective case–control study designs were employed with a prospective logistic regression analysis of data to evaluate the association of two different outcomes (development of CKD and death) with PPI use [8]. The data were obtained from the Veterans Affairs Health Care Upstate New York (VISN2) network, which is composed of five VA medical centers and 29 community based outpatient clinics. The VISN2 database contains longitudinal records of 180,553 VA patients, who were seen in primary care VISN2 clinics from 4/2001 until 4/2008.

### Participants

Patients were included only if they had at least one outpatient estimated glomerular filtration rate (eGFR) estimated by the Chronic Kidney Epidemiology (CKD-EPI) equation during their observation period. For the CKD study, we excluded patients with an eGFR < 60 ml/min/1.73 m^2^ at entry (pre-existing CKD patients). Cases were identified as those patients who were subsequently diagnosed with CKD (ie, had an observed eGFR < 60). Controls were identified as those patients who were not diagnosed with CKD during their observation period. Records were retrospectively reviewed starting from the time of CKD diagnosis for cases, and from the time of last observation for controls to assess whether or not risk factors existed in the history of each patient. For the mortality study, cases were identified as individuals who died during the observation period and controls as individuals who were alive on March 31, 2008.

### Risk factor variables

The primary risk factor to be tested for significance was the prescription of proton pump inhibitor (PPI) in the history of cases or controls. PPI use was determined by whether a prescription was filled during a quarter. In addition to use of PPI (Yes/No), the following control variables were included in the analyses: age, gender, race, time at risk (ie, time from initial observation in the data system to the case–control outcome), and the pre-PPI comorbidity variables: vascular disease, gastrointestinal (GI) comorbidities, chronic obstructive pulmonary disease (COPD), cancer, diabetes, and hypertension.

### Statistical methods

Descriptive baseline statistics were produced for the study population. Patients were divided in 2 groups based on PPI use, Group 1 -on PPI, Group 2 –not on PPI. These two groups were compared using Fisher exact test for categorical variables and t-tests for continuous variables. Similarly descriptive baseline statistics were produced for patients who developed CKD versus those who did not and between patients who died versus those who did not. A prospective logistic analysis of case–control data was used to investigate the association of treatment (exposure to PPI) with each outcome of interest [8]; onset of CKD and mortality, respectively, controlling for age, sex, race, GI and pre-PPI comorbidities. It was shown by Prentice and Pyke that the odds ratio estimators and associated confidence intervals for the effects of risk factor on an outcome variable in case–control studies may be estimated by applying the standard logistic regression modeling to the case–control data as if the data had been obtained in a prospective study. Their work generalizes the findings of Breslow & Powers [9, 10] on the equivalence of odds ratio estimators when both prospective and retrospective logistic models are applied to case–control data. We also performed adjusted stratified analysis by levels of dichotomized versions of the variables in the initial main-effects model, to test for PPI effects within subgroups.

### Sensitivity analysis

We did several sensitivity analyses: 1. We removed individuals with baseline CKD for the analysis of mortality and added CKD (Yes/No) as a covariate in the analysis of mortality outcome; 2. For the second sensitivity analysis, we first defined the propensity score as the estimated probability of receiving PPI, which was obtained from a logistic regression analysis of PPI on age, race, sex and the pre-PPI comorbidity variables: vascular disease, COPD, cancer, diabetes, and hypertension. We repeated our analysis of case–control data for each outcome of interest controlling for propensity score instead of controlling for comorbidities prior to PPI; 3. We also used propensity score matching (PSM) technique to reduce the bias due to confounding variables.

It should be noted, in the sensitivity analyses that involved propensity scoring, all potentially confounding variables except GI were included in the propensity score. GI was of particular interest separately and, therefore, was added to the model directly as a covariate. This allows interpretation of the coefficient on PPI as the effect of PPI unconfounded by preceding GI and the coefficient on GI as the effect of preceding GI that is not mediated through the effect of GI on the decision to prescribe PPI. Thus, since GI is an indication for PPI prescription and it is known that GI is associated with CKD and death, including GI as a covariate directly allows us to assess whether the effect of GI is mediated through its effect on PPI.

All of the analyses were performed using SAS 9.2 (SAS Institute, Cary, NC) [Statistical significance was set to α = 0.05]. The Logistic procedure in SAS was used for the analysis of each model.

## Results

### Patient characteristics

After exclusion of patients with no outpatient eGFR measurement, our sample size was 99,269 individuals. Of these, 36,282 were on PPI at some time during their observation period. 22,807 had CKD at the time of first observation in the VISN2 data system, leaving 76,462 patients for analysis of the CKD onset outcome.

A total of 22,734 were taking PPI (4711 in the case group and 18023 in the control group). Mean age of the patients in the data set for analysis of CKD was 56.6 years. There were 4682 (6.1 %) females and 7719 (10.1 %) African Americans (AA). Table 1 gives the frequencies of risk factors for CKD outcome by PPI status. Patients using PPI were less likely to have vascular disease (15.5 % vs 18.5 %), cancer (7.3 % vs 10.3 %), and diabetes (17.5 % vs 21.2 %) prior to PPI use; and more likely to have COPD (9.85 % vs 8.92 %) or hypertension (62.5 % vs 62.3 %). Logistic regression analysis revealed patients with vascular disease, diabetes, and cancers at baseline were less likely to have received PPI.

**Table 1:** Number and frequencies of risk factors for CKD outcome prior to CKD or end of study time (total of 53,728 individuals not on PPI and total of 22,734 individuals on PPI)

	Not on PPI			On PPI			*p*_value
	Total	Case(CKD)	Control	Total	Case(CKD)	Control	
	53728	14600	39128	22734	4711	18023	
Vascular disease (n)	9948	3703	6245	3525	1046	2479	<0.0001
	18.52 %	25.36 %	15.96 %	15.51 %	22.20 %	13.75 %	
COPD (n)	4792	1541	3251	2239	569	1670	<0.0001
	8.92 %	10.55 %	8.31 %	9.85 %	12.08 %	9.27 %	
Cancer (n)	5540	1689	3851	1648	443	1205	<0.0001
	10.31 %	11.57 %	9.84 %	7.25 %	9.40 %	6.69 %	
Diabetes (n)	11410	4515	6895	3976	1206	2770	<0.0001
	21.24 %	30.92 %	17.62 %	17.49 %	25.60 %	15.37 %	
Hypertension (n)	33496	12024	21472	14202	3734	10468	<0.0001
	62.34 %	82.36 %	54.88 %	62.47 %	79.26 %	58.08 %	
GI (n)	7494	1913	5581	12832	2324	10508	<0.0001
	13.95	13.1	14.26	56.44	49.33	58.3	
Black (n)	5404	1067	4337	2315	357	1958	0.5994
	10.06 %	7.31 %	11.08 %	10.18 %	7.58 %	10.86 %	
Female (n)	3340	534	2806	1342	214	1128	0.099
	6.22 %	3.66 %	7.17 %	5.90 %	4.54 %	6.25 %	
Age in years (mean)	56.94	66.9	53.2	56.3	63.88	54.32	<0.0001
(STD)	15.38	11	15.2	13.17	10.9	13	
Time at risk (quarters) (mean)	10.37	6.55	11.8	14.1	10.83	14.95	<0.0001
(STD)	7.66	5.9	7.74	7.1	6.17	7.08	

Out of the 99,269 patients in the mortality study, 36,282 were taking PPI at some time during their observation period. Table 2 gives the frequencies of risk factors in the mortality outcome study by PPI status. In the data set for analysis of mortality, mean age of the patients was 60.6 years. There were 5437 (5.5 %) female and 8921 (9.0 %) African Americans.

**Table 2:** Frequencies of risk factors for mortality outcome prior to end of study time (total of 62,987 individuals not on PPI and total of 36,282 individuals on PPI)

	Not on PPI			On PPI			*p*_value
	Total	Case (death)	Control	Total	Case (death)	Control	
	62987	6897	56090	36282	4861	31421	
Vascular disease (n)	16806	3257	13549	8683	1982	6701	<0.0001
	26.68 %	47.22 %	24.16 %	23.93 %	40.77 %	21.33 %	
COPD (n)	6856	1696	5160	4298	1116	3182	<0.0001
	10.88 %	24.59 %	9.20 %	11.85 %	22.96 %	10.13 %	
Cancer (n)	9220	1954	7266	3749	1073	2676	<0.0001
	14.64 %	28.33 %	12.95 %	10.33 %	22.07 %	9.52 %	
Diabetes (n)	15526	2295	13231	8633	1642	6991	<0.0001
	24.65 %	33.28 %	23.59 %	23.79 %	33.78 %	22.25 %	
Hypertension (n)	42625	5756	36869	26503	4231	22272	<0.0001
	67.67 %	83.46 %	65.73 %	73.05 %	87.04 %	70.88 %	
GI (n)	9274	1263	8011	20141	2227	17914	<0.0001
	14.72 %	18.31 %	14.28 %	55.51 %	45.81 %	57.01 %	
Black (n)	5768	489	5279	3153	362	2791	0.0132
	9.16 %	7.09 %	9.41 %	8.69 %	7.45 %	8.88 %	
Female (n)	3582	122	3460	1855	109	1746	<0.0001
	5.69 %	1.77 %	6.17 %	5.11 %	2.24 %	5.56 %	
Age in years(mean)	59.9	72.87	58.34	61.7	70.28	60.37	<0.0001
(STD)	16.12	11.2	15.9	14.1	11.55	14	
Time at risk (quarters) (mean)	14.13	11.13	14.5	17.3	14.5	17.72	<0.0001
(STD)	7.63	6.11	7.7	6.64	5.8	6.65	

Note: Given *p*-values are for PPI group differences. For categorical variables, the proportions of patients with the characteristics in two PPI groups were compared using Fisher exact test. We performed a simple *t* test analysis, with adjustment for unequal variances when appropriate, to compare the means of continuous variables

### Multivariate analysis

**Fig. 1:**
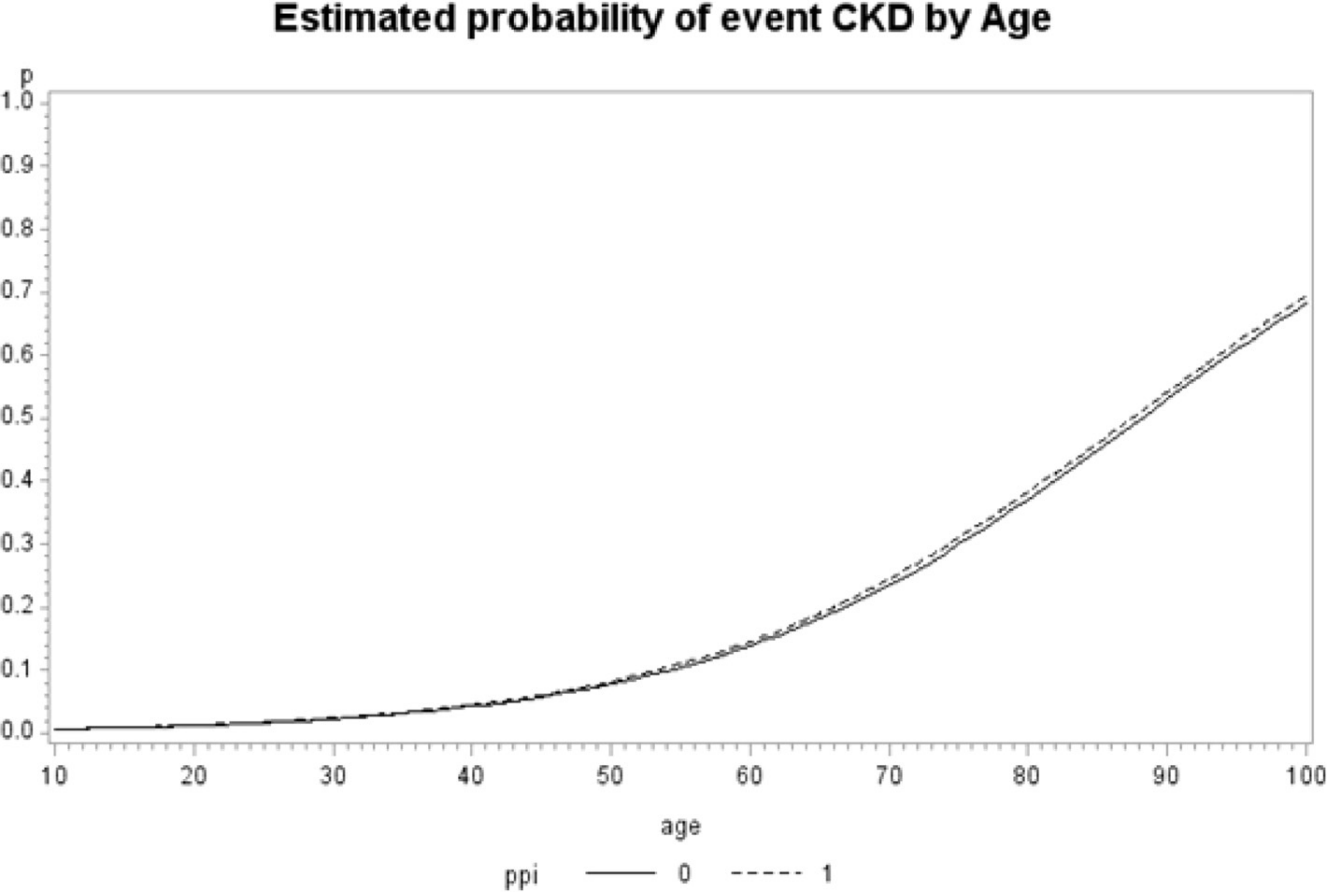
Estimated probability of CKD by age from the fitted model when interaction of PPI and Age is added to the model for CKD outcome

**Fig. 2:**
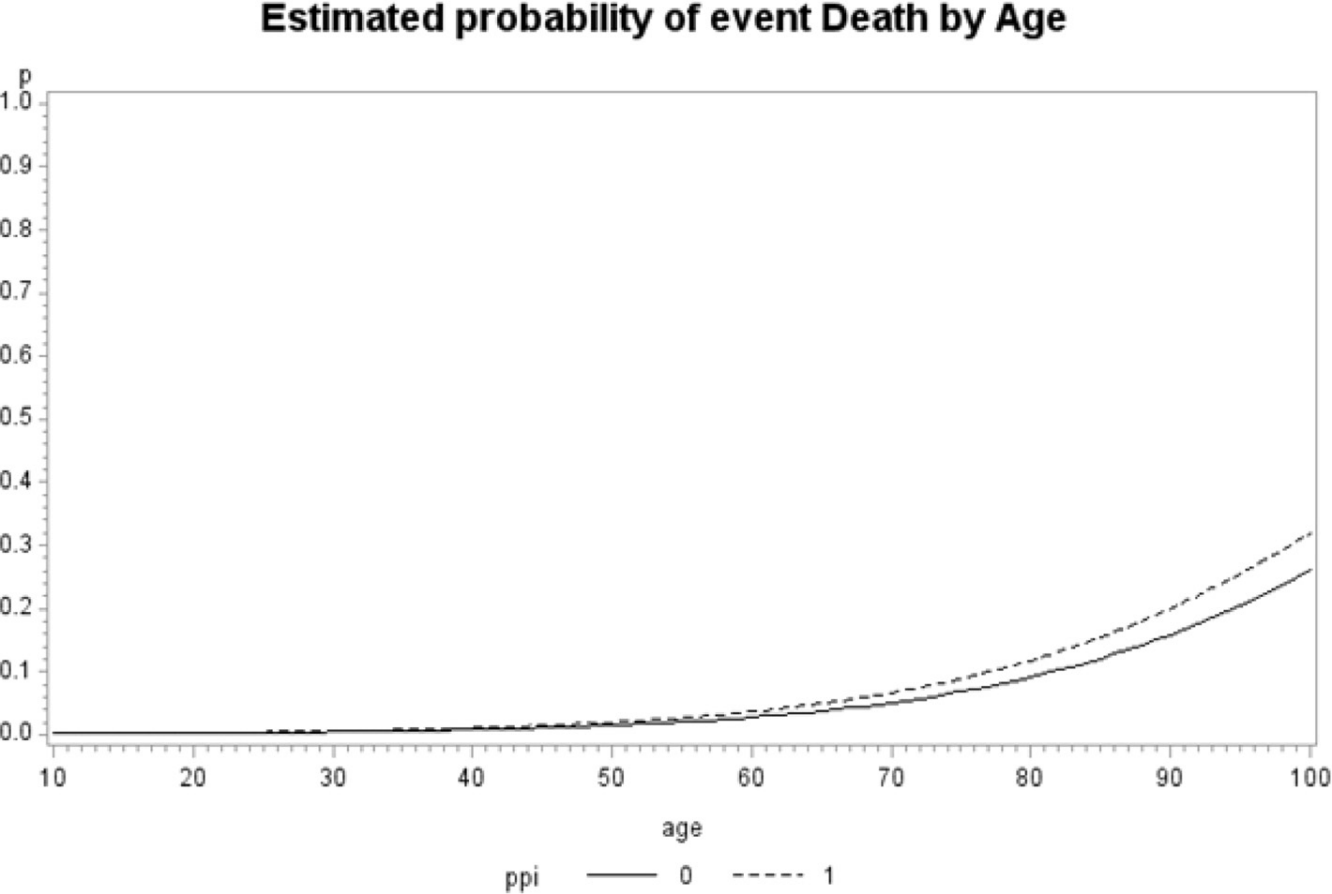
Estimated probability of death by age, from the fitted model when interaction of PPI and Age is added to the model for mortality

Logistic analyses revealed a statistically significant increase in the rate of occurrence of mortality and CKD among patients who were taking PPIs compared to those who were not taking PPIs (Table 3). Figures 1 and 2 give the estimated probabilities of event by age for CKD and mortality analysis. We estimated probability of event by age from the fitted model (mean time at risk were 12.4 quarters and 15.9 for CKD and mortality, respectively). There was a significant effect of the interaction of age and PPI use (*p*-value <0.0001), in models for both development of CKD and mortality. The result shows patients younger than 53 years old were significantly at higher risk of CKD incidence if taking PPI. Patients younger than 78 years old had significantly in higher risk of death if taking PPI. To determine whether the effect of PPI varied according to baseline characteristics, we performed stratified analyses for the risk of CKD and mortality. Patients who were white, male, <65 years and did not have DM, vascular disease, cancer were at greater risk of CKD outcome if on PPI than if not on PPI blockers. However mortality outcome with PPI did not vary based on demographic or comorbidity (Table 4).

**Table 3:** Estimate of odds ratios, with the 95 % confidence limits

		For Mortality outcome Odds Ratio Estimates				For CKD outcome Odds Ratio Estimates			
Effect	Contract	Point Estimate	95 % Wald Confidence Limits		*p*-value	Point Estimate	95 % Wald Confidence Limits		*p*-value
PPI	Yes vs No	1.76	1.68	1.84	<.0001	1.10	1.05	1.16	<.0001
age	1 year increase	1.07	1.06	1.07	<.0001	1.07	1.07	1.07	<.0001
Race	Black vs White	1.41	1.30	1.53	<.0001	0.92	0.86	0.99	0.0269
Sex	Female vs Male	0.62	0.54	0.72	<.0001	1.32	1.20	1.45	<.0001
Vascular Disease	Yes vs No	1.52	1.45	1.59	<.0001	0.94	0.89	0.98	0.009
COPD	Yes vs No	2.41	2.28	2.54	<.0001	0.97	0.91	1.04	0.378
Cancer	Yes vs No	1.91	1.82	2.02	<.0001	0.78	0.74	0.84	<.0001
Diabetes	Yes vs No	1.53	1.46	1.61	<.0001	1.66	1.59	1.74	<.0001
Hypertension	Yes vs No	1.38	1.30	1.47	<.0001	2.43	2.31	2.55	<.0001
GI	Yes vs No	1.03	0.98	1.08	0.25	0.99	0.94	1.04	0.6208
Time at risk	1 quarter increase	0.91	0.91	0.91	<.0001	0.90	0.89	0.90	<.0001

**Table 4:** Adjusted OD and 95 % confidence interval for CKD and Mortality outcomes associated with PPI for each subgroups

Subgroup		OR	CKD		Mortality	95%CI	
			95%CI		OR		
Age	<65	1.24	1.17	1.29	2.26	2.07	2.47
	>65	1.21	0.89	1.69	1.60	1.51	1.70
Gender	Female	1.09	0.94	1.49	1.67	1.24	2.30
	Male	1.10	1.05	1.15	1.70	1.68	1.85
Race	Black	1.01	0.85	1.18	1.68	1.41	2.00
	White	1.16	1.06	1.17	1.77	1.68	1.86
GI	Absent	1.21	1.14	1.29	2.18	2.00	2.24
	Present	0.93	0.86	1.10	1.18	1.08	1.28
DM	Absent	1.11	1.05	1.17	1.70	1.60	1.80
	Present	1.07	0.99	1.18	1.89	1.74	2.06
HTN	Absent	1.21	1.09	1.34	1.63	1.44	1.85
	Present	1.07	1.01	1.12	1.78	1.69	1.87
Vascular	Absent	1.13	1.07	1.19	1.87	1.76	1.99
	Present	1.02	0.93	1.13	1.63	1.51	1.75
Cancer	Absent	1.10	1.05	1.16	1.70	1.61	1.80
	Present	1.11	0.96	1.28	1.90	1.72	2.11

### Sensitivity analyses

1 Adding CKD (Yes/No) as a covariate in the analysis of mortality, the CKD effect was significant but the PPI effect on mortality did not change; 2. When we controlled for propensity score the odds ratio for CKD outcome was 1.08 (95 % CI 1.03–1.13), and for mortality outcome the odds ratio was 1.70 (95 % CI 1.62–1.79), for PPI versus no PPI. 3. For the propensity matched data results were similar.

## Discussion

Our study revealed that PPI use was associated with increased odds of development of CKD and death. Although the association of PPI use with mortality has been widely reported, we found an association of PPI use with development of CKD. It is not surprising that PPI use is associated with CKD as these drugs are one of the most common causes of AIN in the United States [11].

Our study showed that PPI use increased the odds of development of CKD by 10 %. The most likely explanation is unrecognized or partially recovered AIN. Thirty to 70 % of patients with drug induced AIN do not fully recover their baseline renal function, likely due to rapid transformation of interstitial cellular infiltrates into large areas of fibrosis [12]. Simpson et al found an incidence of AIN of 8.0 per 100,000 person-years (95 % CI 2.6–18.7), based on 15 cases and an estimated 750,000 1-month treatments dispensed annually [13]. Blank et al found similar results in a case control study [14]. Recovery from AKI occurs following withdrawal of the offending drug with or without corticosteroid treatment may not be complete and many patients are left with some degree of renal impairment [11]. Geevasinga et al identified 18 cases of biopsy-proven PPI-induced AIN causing AKI in a retrospective case review. All patients recovered renal function, but the mean calculated creatinine clearance was 15.9 ml/min/ 1.73 m^2^ and 11.5 ml/min/1.73 m^2^ lower than baseline at 3 and 6 months, respectively [15]. Simpson et al also noted an incomplete recovery of renal function after PPI induced AIN [13].

Failure to recognize this entity early in the course may lead to irreversible interstitial fibrosis and CKD. Thus an early diagnosis and withdrawal of the offending drug is the key to prevent potentially life threatening renal failure. However, this may be difficult because most of these patients have nonspecific symptoms on presentation. In a systematic review by Sierra et al including 60 cases of PPI use associated AIN, 12–30 % patients had nonspecific symptoms and 8 % of patients were asymptomatic. Pyuria was present in 61 % and eosinophiluria in only 21 % of cases [16]. Only about 10 % of patients with PPI induced AIN presented with the classic triad of fever, rash and eosinophilia [11]. Most of these patients had insidious onset of AKI [13].

Similar results were shown recently by Xie et al [17] and Lazarus et al [18]. Lazarus et al studied in 2 data cohort (Atherosclerosis Risk in Communities (ARIC) and Geisinger Health system replication cohort) and found that PPI were associated with higher odds for CKD. In the ARIC cohort use of PPI was defined by self-reported use and CKD was defined using ICD codes, which had a specificity of 35.5 %. In the Geisinger Health system replication cohort, the diagnosis of CKD was based on eGFR and use of PPI was based on prescription. Both these cohorts had higher cardiovascular comorbidities in PPI users (in our cohort PPI users had less cardiovascular comorbidity) [18]. Higher odds of CKD (1.3–1.5) compared to our cohort (1.10) may be explained by higher comorbidity and longer duration of follow up in these cohorts.

In our study we found that younger individuals were more likely to develop CKD associated with PPI use. This is in contrast to the observation in Sierra’s systematic review of AIN who found that AIN due to PPI was more common among older patients. Although older patients are more likely to have multiple comorbidities and the renal interstitium may be more vulnerable to damage due to compromised blood flow, allowing a larger exposure time to the medications [13], PPI use was associated with increased odds of CKD among younger patients in our study. It is difficult to speculate the mechanism among younger patients. It is possible that the prevalence of CKD among elderly is high with or without PPI, but in the younger population the prevalence of CKD without PPI use is quite low making the prevalence of CKD associated with PPI use more significant. In a similar phenomenon prevalence of cardiovascular comorbidity in dialysis patients is more than 100 times higher in a younger population compared to only 2 times higher at the age of 80 years as compared to the general population [19]. Another potential mechanism by which these medications can lead to CKD is through progressive chronic interstitial nephritis due to magnesium deficiency. Hypomagnesemia has been shown to cause endothelial cell dysfunction, inflammation and oxidative stress [20, 21]. Also, there is evidence of association between hypomagnesemia and albuminuria in patients with type 2 diabetes [22]. Pham et al reported that serum magnesium level was significantly associated with the slope of inverse serum creatinine in type 2 diabetic patients with near normal renal function [23]. Sakaguchi et al, in a retrospective cohort study found that hypomagnesemia was significantly associated with progression to end stage renal disease (ESRD) in patients with type 2 diabetic nephropathy but not in those with nondiabetic CKD [24]. Rats fed a magnesium deficient diet have been shown to have higher urine N-acetyl-β-D-glucosaminidase levels, reflecting that magnesium deficiency induces renal interstitial tubular injury [25]. We did not examine the magnesium levels of these patients, thus additional studies are needed to explore any substantial association between PPI induced hypomagnesemia and chronic kidney disease. The precise pathogenesis of chronic kidney disease in patients on proton pump inhibitors needs to be further investigated.

Our study showed that the use of PPIs is associated with a 75 % increased risk of mortality. Other studies have also shown a similar association of PPI use and increased risk of death, especially in elderly and institutionalized patients and among patients who were on clopidogrel [4, 6].

There are a few limitations to this study. There is potential for indication bias in our results. However, indication bias in this cohort is unlikely. In fact, the study patients on PPI were less likely to have a higher comorbidity burden than those who did not receive PPI. This actually strengthens the case for the association of PPI use with CKD and mortality. Covariates that are not associated with either treatment or outcome do not cause a bias. The covariates that are associated with both treatment and outcome were included in the model and, hence, were controlled for. It is possible that variables not in our data set, such as use of NSAIDs, smoking and therefore not controlled for could cause indication bias, as is true in all observational studies. Furthermore we also did propensity score analyses to mitigate indication bias, and the results didn’t change. However, we note the potential for indication bias as a limitation of any observational study. We used one eGFR less than 60 ml/min/1.73 m^2^ as the definition of CKD, because although this does not meet the KDOQI definition, most large epidemiologic studies have used a single eGFR definition for CKD. Our results may not be generalizable due to the fact that females and non-whites are underrepresented in the VA study population. However, this was a real world cohort from the largest integrated health care system in United States and utilizes a uniform data collection system.

## Conclusions

In this study, we found that long-term use of proton pump inhibitors is associated with increased risk of development of CKD and death. Although cause and effect cannot be determined with an observational study, with the large number of patients being treated with proton pump inhibitors, healthcare providers need to be cautious in prescribing these drugs because of the potential side effects.

## Abbreviations

AA, African Americans; AIN, acute interstitial nephritis; ARIC, Atherosclerosis Risk in Communities; CKD, chronic kidney disease; CKD-EPI, Chronic Kidney Disease Epidemiology Collaboration; COPD, chronic obstructive pulmonary disease; eGFR, estimated glomerular filtration rate; GERD, gastroesophageal reflux disease; GI, gastrointestinal; NSIADs, non-steroidal anti-inflammatory drugs; PPIs, proton pump inhibitors.
